# Aspartame Cancer Risks Revisited: Prenatal Exposure May Be Greatest Concern

**Published:** 2007-09

**Authors:** M. Nathaniel Mead

Aspartame is an artificial sweetener used in more than 6,000 diet products, beverages, and pharmaceuticals. In March 2006, *EHP* published the first compelling experimental evidence for the carcinogenic effects of aspartame at a dose level within range of human daily intake **[*EHP* 114:379–385; Soffritti et al.]**. A second animal study by the same research team now indicates that the carcinogenic effects of aspartame are magnified when exposure begins during fetal life **[*EHP* 115:1293–1297; Soffritti et al.]**.

The first study involved a much larger sample size than had been used in previous experiments. It showed a dose-related increase in the incidence of various malignant tumors in female rats fed aspartame from 8 weeks of age until natural death. The experiment was impressive for its long observation period and comprehensive assessment of aspartame’s carcinogenic potential. Nevertheless, as the researchers acknowledged, the study did not take into account prenatal or perinatal exposures.

In the new study, the investigators added aspartame to the standard diets of male and female Sprague-Dawley rats from the twelfth day of fetal life until natural death. Rats were fed in groups of 70 to 95 each at aspartame concentrations of 2,000, 400, and 0 ppm—approximately equivalent to a daily intake of 100, 20, or 0 mg/kg body weight. The current limits for acceptable daily intake are set at 50 mg/kg body weight in the United States and 40 mg/kg body weight in Europe.

The researchers report that rats fed at the 400 ppm level showed nonsignificant increases in malignancies. For animals fed at the 2,000 ppm level, there was a significant increase in the incidence of lymphomas/leukemias and malignant mammary tumors. Furthermore, compared with the team’s earlier study in which animals were dosed postnatally only, the incidence of animals bearing lymphomas/leukemias increased from 18.7% to 31.4%. The 2,000 ppm level corresponds to an assumed daily intake of 100 mg/kg body weight—approximately the equivalent of a 45-pound child drinking 5 cans of diet soda or a 150-pound adult consuming 14 packets of sweetener per day.

Although recent epidemiologic studies have not found an association between aspartame and human cancers, those studies were not designed to measure cancer risks associated with fetal exposures. The public health implications of the new findings are considerable. Currently, more than 200 million people regularly consume aspartame, and children and women of childbearing age (which presumably includes many who are pregnant and breastfeeding) are among the major consumers. If the U.S. FDA were to conclude that exposure to aspartame causes cancer in rodents, the agency would be required by law to revoke its approval for the popular sweetener.

